# Eye Movement-Related Confounds in Neural Decoding of Visual Working Memory Representations

**DOI:** 10.1523/ENEURO.0401-17.2018

**Published:** 2018-10-10

**Authors:** Pim Mostert, Anke Marit Albers, Loek Brinkman, Larisa Todorova, Peter Kok, Floris P. de Lange

**Affiliations:** 1 Donders Institute for Brain, Cognition and Behaviour, Radboud University, Nijmegen 6500 HB, The Netherlands; 2 Abteilung Allgemeine Psychologie, Justus-Liebig-Universität, Giessen 35394, Germany; 3 Department of Psychology, Utrecht University, Utrecht 3584 CS, The Netherlands; 4 Center for Mind/Brain Sciences, University of Trento, Rovereto TN 38068, Italy; 5 Princeton Neuroscience Institute, Princeton University, Princeton, NJ 08544; 6 Department of Psychology, Yale University, New Haven, CT 06511

**Keywords:** eye movements, magnetoencephalography, multivariate decoding, visual working memory

## Abstract

A relatively new analysis technique, known as neural decoding or multivariate pattern analysis (MVPA), has become increasingly popular for cognitive neuroimaging studies over recent years. These techniques promise to uncover the representational contents of neural signals, as well as the underlying code and the dynamic profile thereof. A field in which these techniques have led to novel insights in particular is that of visual working memory (VWM). In the present study, we subjected human volunteers to a combined VWM/imagery task while recording their neural signals using magnetoencephalography (MEG). We applied multivariate decoding analyses to uncover the temporal profile underlying the neural representations of the memorized item. Analysis of gaze position however revealed that our results were contaminated by systematic eye movements, suggesting that the MEG decoding results from our originally planned analyses were confounded. In addition to the eye movement analyses, we also present the original analyses to highlight how these might have readily led to invalid conclusions. Finally, we demonstrate a potential remedy, whereby we train the decoders on a functional localizer that was specifically designed to target bottom-up sensory signals and as such avoids eye movements. We conclude by arguing for more awareness of the potentially pervasive and ubiquitous effects of eye movement-related confounds.

## Significance Statement

Neural decoding is an important and relatively novel technique that has opened up new avenues for cognitive neuroscience research. However, with its promises also come potential caveats. In this study we show that neural decoding may be susceptible to confounds induced by small task- and stimulus-specific eye movements in the context of a visual working memory (VWM) task. Such eye movements during working memory tasks have been reported before and may in fact be a common phenomenon. Given the widespread use of neural decoding and the potentially contaminating effects of eye movements, we therefore believe that our results are of significant relevance for the field.

## Introduction

Neural decoding, or multivariate pattern analysis (MVPA), is a popular analysis technique that has obtained considerable momentum in the field of cognitive neuroimaging (Haxby et al., 2014; Grootswagers et al., 2017). It refers to uncovering a factor of interest, for instance stimulus identity, from multivariate patterns in neural signals such as those measured by magnetoencephalography (MEG) or functional magnetic resonance imaging (fMRI). Decoding allows one to probe the representational contents of a neural signal, rather than overall activity levels, with superior sensitivity. However, this sensitivity may require extra vigilance at the end of the user, because these analyses may also be particularly sensitive to potentially confounding factors. Here we demonstrate such an example, specifically in the context of visual working memory (VWM), where a decoding analysis is contaminated by stimulus-specific eye movements. Given the widespread use of these techniques and its pivotal contributions to contemporary VWM theories, we argue that appreciation of these potential caveats is important.

VWM is the ability to retain and use visual information about the world for a short period of time, even when the original external source of that information is no longer available. Neural decoding has been frequently applied in the study of VWM to elucidate where, when and how a memorandum is encoded in the brain. This was first demonstrated by Harrison and Tong (2009) and Serences et al. (2009), who were able to decode the orientation of a memorized grating from visual cortex. Further VWM decoding studies extended Harrison and Tong (2009)’s paradigm in varying ways to study, among others, mental imagery, mental transformations, and spatial working memory (Albers et al., 2013; Christophel et al., 2015, 2017; Foster et al., 2016; Gayet et al., 2017). The paradigm has also been ported to electrophysiological studies using MEG or electroencephalography to capitalize on the high temporal resolution offered by those methods (Wolff et al., 2015, 2017; Foster et al., 2016; King et al., 2016). These results have led to important new theories, among others the idea that high-fidelity VWM representations are stored in early sensory cortex (Albers et al., 2013; Sreenivasan et al., 2014), the activity-silent coding hypothesis (Stokes, 2015; Wolff et al., 2015, 2017; Rose et al., 2016; Rademaker and Serences, 2017) and the dynamic coding framework (Stokes et al., 2013, 2015; King et al., 2016; Spaak et al., 2017).

In the current study, human volunteers performed a combined VWM/imagery task, while we traced the representational contents of their neural activity as measured by MEG. The experiment was designed to elucidate the temporal profile of the memorized item's neural representation. However, control analyses revealed that our data were severely contaminated by small eye movements. In this article, we first describe the eye movement analysis to show how the identity of the memorized item could be decoded from gaze position. Next, we present the naive results as they would have been, had we not been aware of the confound. This highlights how these could easily have been mistaken to provide genuine insight into the neural mechanisms underlying VWM. Finally, we present a potential solution by training the decoders on separate functional localizer blocks, which allowed us to extract the sensory-specific neural patterns, thereby effectively bypassing the eye-movements confounds.

## Materials and Methods

### Subjects

Thirty-six human volunteers were recruited from the local institute’s subject pool to participate in a behavioral screening session. Of these, 24 (13 male; mean age: 26.8 years, range: 18–60) were selected to participate in the MEG experiment (see below, Experimental design and procedure). Of these 24 selected subjects, three were excluded from MEG analysis due to poor data quality and another four were excluded from the analyses regarding eye movements, because the eye-tracker failed to track the eye reliably in those subjects. The experiment was approved by the local ethics committee and conducted according to the guidelines set out by the committee. All participants provided written informed consent and received either monetary compensation or course credits.

### Stimuli

Stimulation was visual and consisted of sinusoidal gratings with a spatial frequency of 1 cycle/°, 80% contrast and one random phase per experimental block. The gratings were masked at an outer radius of 7.5° and an inner aperture radius of 0.7° and presented on a gray background (luminance: 186 cd/m^2^). Stimuli were generated and presented using MATLAB with the Psychtoolbox extension (Kleiner et al., 2007).

### Experimental design and procedure

**Figure 1.**
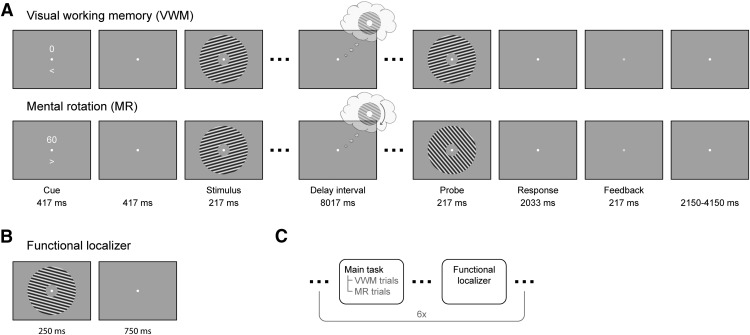
Experimental paradigm. ***A***, In the combined VWM/imagery blocks, subjects were instructed to vividly imagine a grating and to either keep that in mind (VWM condition) or rotate it mentally over a cued number of degrees (MR condition). ***B***, In the functional localizer block, oriented gratings were continuously presented while the subject’s attention was drawn to a task at fixation. ***C***, The VWM/imagery and localizer blocks were performed in alternating order.

The main task was to vividly imagine and remember an oriented grating and, in some conditions, mentally rotate this grating over a certain angle. Each trial began with a dual cue that indicated both the amount (presented above fixation) and the direction (> for clockwise and < for counterclockwise, presented below fixation) of mental rotation (MR) that was to be performed in that trial (Fig. 1). The amount could be either 0°, 60°, 120°, or 180°, in either clockwise or counterclockwise direction, where 0° corresponded to a VWM task. This condition will henceforth be referred to as the VWM condition, and the other three conditions, which corresponded to imagery, as the MR conditions. This cue lasted for 417 ms, after which a blank screen was shown for another 417 ms. A fixation dot (four pixels in diameter) was present throughout the entire trial, and throughout the entire block. After the blank, a grating was presented for 217 ms that could have either of three orientations: 15°, 75°, or 135° (clockwise with respect to vertical). Next, a blank delay period of 8017 ms followed, during which subjects were required to keep the starting grating in mind and, in a subset of trials, mentally rotate it. The delay period was terminated by the presentation of a probe grating for 217 ms, whose orientation was slightly jittered (see below, Staircase procedure) with respect to the orientation that subjects were supposed to have in mind at that moment. Subjects then indicated with a button press whether the probe was oriented clockwise or counterclockwise relative to their internal image. The response period lasted until 2033 ms after probe, after which feedback was given. There were three trials per design cell (four arcs of rotation and two directions) per block, resulting in 24 trials per block. In addition, there were two catch trials per block, in which the probe grating was presented at an earlier moment in the delay interval to gauge ongoing rotation. All trials were presented in pseudorandomized order. The catch trials were excluded from further analysis, because subjects indicated to find them difficult and confusing. In general, each experiment consisted of six experimental blocks (although some subjects performed five, seven, or eight blocks), preceded by one or more practice blocks, resulting in a total of 144 experimental trials for most of the participants.

Interleaved with the VWM/imagery blocks, there were six functional localizer blocks (Fig. 1*B*,*C*). In these blocks, gratings of six different orientations (15° to 165°, in steps of 30°) were presented for 250 ms with an intertrial interval of 750 ms. Each block consisted of 120 trials, resulting in a total of 120 trials per orientation. The task was to press a button when a brief flicker of the fixation dot occurred. Such a flicker occurred between 8 and 12 times (randomly selected number) per block, at random times. Using such a task we ensured that spatial attention was drawn away from the gratings while stimulating subjects to maintain fixation, allowing us to record activity that predominantly reflected bottom-up, sensory-specific signals (Mostert et al., 2015).

Before the MEG session, the volunteers participated in a behavioral screening session that served to both train the subjects on the task as well as to assess their ability to perform it. Subjects were instructed to mentally rotate the stimulus at an angular velocity of 30°/s by demonstrating examples of rotations on the screen. Moreover, during this session subjects were required to press a button as soon as they achieved a vivid imagination of the grating on completion of the cued rotation. This provided a proxy of the speed at which they actually performed the rotation and was used as selection criterion for participation in the MEG session.

### Staircase procedure

The amount of jitter of the probe grating was determined online using an adaptive staircasing procedure to equalize subjective task difficulty across conditions and subjects. The starting difference was set to 15° and was increased by 1° following an incorrect response and decreased by 0.5488 after two consecutive correct responses. Such a procedure has a theoretical target performance of ∼80% correct (García-Pérez, 1998). Four separate staircases were used, one for each of the VWM and MR conditions.

### MEG recordings, eye-tracker recordings, and pre-processing

Neural activity was measured using a whole-head MEG system with 275 axial gradiometers (VSM/CTF Systems) situated in a magnetically shielded room. A projector outside the room projected via a mirror system onto the screen located in front of the subject. Fiducial coils positioned on the nasion and in the ears allowed for online monitoring of head position and for correction in between blocks if necessary. Both vertical and horizontal electrooculogram (EOG) as well as electrocardiogram were obtained to aid in the recognition of artifacts. All signals were sampled at 1200 Hz and analyzed offline using the FieldTrip toolbox (Oostenveld et al., 2011). The data were notch-filtered at 50 Hz and corresponding harmonics to remove line noise, and subsequently inspected in a semi-automatic manner to identify irregular artifacts. After rejection of bad segments, independent component analysis was used to remove components that corresponded to regular artifacts such as heartbeat, blinks and eye movements (although our results suggest that the removal of eye movement-related artifacts was imperfect, see Results and Discussion). The cleaned data were baseline-corrected on the interval of -200–0 ms, relative to stimulus onset.

Gaze position and pupil dilation were continuously tracked throughout the experiment using an Eyelink 1000 (SR Researcher) eye-tracker. The eye-tracker was calibrated before each session and signals were sampled at 1200 Hz. Because we were interested in eye-movements induced by the experimental stimulation, we removed any slow drifts in the signal by baseline-correcting the signal on an interval of -200–0 ms relative to cue onset.

### Data sharing

All data, as well as analysis scripts required to obtain the presented figures, are available from the Donders Institute for Brain, Cognition, and Behavior repository at http://hdl.handle.net/11633/di.dccn.DSC_3018016.04_526.

### Classification and decoding analyses

Originally, we first focused on the neural data. Broadly, we conducted two lines of decoding analyses. In the first, we focused only on the blocks in which participants performed the combined VWM/imagery task, using 8-fold cross-validation. We trained a three-class probabilistic classifier that returns the probability that a given trial belongs to either of the three presented grating orientations. To improve signal-to-noise ratio, yet retain the ability to draw firm conclusions regarding the timing of any decoded signal, we smoothed the data using a moving average with a window of 100 ms. The classifier was trained across the spatial dimension (i.e., using sensors as features), on trials from all conditions (i.e., all amounts and directions of rotation). This may seem counterproductive, because the mental contents diverge over the delay interval and there should therefore be no systematic relationship between the MEG data and the stimulus label. However, our rationale was that regardless of condition, subjects need to first perceive, encode and maintain the presented stimulus before they can even commence the task, be it VWM or MR. Thus, we expected to be able to extract the neural pattern of the presented stimulus during at least the physical presentation and a brief moment after that. This classifier was then trained and applied across all time points, resulting in a temporal generalization matrix (King and Dehaene, 2014). It is important to note that we trained the classifiers only using the labels of the presented stimulus but sorted the data in varying ways when testing the performance. For example, by looking at an early training time point, but a late testing time point, we tested whether we could decode the orientation of the grating kept in mind near the end of the delay period, on the basis of the pattern evoked by the presented stimulus early in the trial.

In the second line of analysis, we trained a continuous orientation decoder on the functional localizer. The larger number of orientations sampled in the functional localizer allowed us to decode a continuous estimate of represented orientation, rather than a discrete one from a fixed number of classes. We applied this decoder to the VWM/imagery task and subsequently related the decoded orientation to the true presented orientation by calculating a quantity intuitively similar to a correlation coefficient (see below, Continuous orientation decoder). Here too, we extended the procedure to include all pairwise training and testing time points, resulting in temporal generalization matrices (King and Dehaene, 2014).

In the control analysis, where we tested for a systematic relationship between gaze position and VWM contents, we repeated the first line of analysis described above, but instead used the gaze position (*x*- and *y*-coordinates) as features rather than the MEG data.

### Multi-class probabilistic classifier

The three-class classifier was based on Bishop (2006, pp 196–199). Briefly, the class-conditional densities were modeled as Gaussian distributions with assumed equal covariance. By means of Bayes’ theorem, and assuming a flat prior, this model was inverted to yield the posterior probabilities, given the data. Specifically, let **x** be a column vector with length equal to the number of features [number of sensors for MEG data, two for gaze position (horizontal and vertical location)] containing the data to be classified, then the posterior probability that the data belongs to class *k* is given by the following equations:

$$P\left(class=k\right|x)=\frac{\exp\left(a_{k}\right)}{\sum_{j}exp(a_{j})}$$

$$a_{k}\left(x\right)=w_{k}^{T}x+w_{k0}$$

$$w_{k}=S^{-1}m_{k}$$

$$w_{k0}=-\frac{1}{2}m_{k}^{T}S^{-1}m_{k}$$

where **m***_k_* is the mean of class *k* and **S** is the common covariance, both obtained from the training set. The latter was calculated as the unweighted mean of the three covariance matrices for each individual class, and subsequently regularized using shrinkage (Blankertz et al., 2011) with a regularization parameter of 0.05 for the MEG data and 0.01 for the eye-tracker analysis.

### Continuous orientation decoder

The continuous orientation decoder was based on the forward-modeling approach as described in Brouwer and Heeger (2009, 2011) but adapted for improved performance (Kok et al., 2017). The forward model postulates that a grating with a particular orientation activates a number of hypothetical orientation channels, according to a characteristic tuning curve, that subsequently lead to the measured MEG data. We formulated a model with 24 channels spaced equally around the circle, whose tuning curves were governed by a Von Mises curve with a concentration parameter of 5. Note that all circular quantities in the analyses were multiplied by two, because the formulas we used operate on input that is periodic over a range of 360° but grating orientation only ranges from 0° to 180°. Next, we inverted the forward model to obtain an inverse model. This model reconstructs activity of the orientation channels, given some test data. In this step we departed from Brouwer and Heeger (2009, 2011)’s original formulation in two aspects. First, we estimated each channel independently from each other, allowing us to include more channels than there are stimulus classes. Second, we explicitly took into account the correlational structure of the noise, which is a prominent characteristic of MEG data, to improve decoding performance (Blankertz et al., 2011; Mostert et al., 2015). For full implementational details, see Kok et al. (2017). The decoding analysis yields a vector **c** of length equal to number of channels (24 in our case) with the estimated channel activity in a test trial, for each pairwise training and testing time point. These channels activities were then transformed into a single orientation estimate *θ* by calculating the circular mean (Berens, 2009) across all the orientations the channels are tuned for, weighted by each individual activation:

$$\theta =arg\left[\sum_{j}c_{j}exp(i\mu_{j})\right]$$

where the summation is over channels, *µ_j_* is the orientation around which the *j*th channel tuning curve is centered, and *i* is the imaginary unit. These decoded orientations can then be related to the true orientation, across trials, as follows:

$$z=\frac{1}{N}\sum_{k}^{N}exp\left[i\left(\theta_{k}-\varphi_{k}\right)\right]$$

$$\rho =|z|cos(\arg\left(z\right))$$

where *N* is the number of trials and *φ_k_* is the true orientation on trial *k*. The quantity *ρ* is also known as the test statistic in the V test for circular uniformity, where the orientation under the alternative hypothesis is prespecified (Berens, 2009). This quantity has properties that make it intuitively similar to a correlation coefficient: it is +1 when decoded and true orientations are exactly equal, -1 when they are in perfect counterphase and 0 when there is no systematic relationship or when they are perfectly orthogonal.

### Statistical testing

All inferential statistics were performed by means of a permutation test with cluster-based multiple comparisons correction (Maris and Oostenveld, 2007). These were applied to either whole temporal generalization matrices, or horizontal cross-sections thereof (i.e., a fixed training window). These matrices/cross-sections were tested against chance-level (33%) in the classification analysis, or against zero in the continuous decoding analysis. In the first step of each permutation, clusters were defined by adjacent points that crossed a threshold of *p* < 0.05 according to a two-tailed one-sample *t* test. The *t* values were summed within each cluster, but separately for positive and negative clusters, and the largest of these were included in the permutation distributions. A cluster in the true data were considered significant if its *p* value was <0.05. For each test, 10,000 permutations were conducted.

### Spatial patterns and source analysis

To interpret the signals that the classifier and decoder pick up, we looked at the corresponding spatial patterns (Haufe et al., 2014). The spatial pattern is the signal that would be measured if the latent variable that is being decoded is varied by one unit. For both the probabilistic classifier and the continuous orientation decoder, this comes down to the difference ERF between each category and the average across all categories. This yields one spatial pattern for each class, and these were subsequently averaged across classes, as well as across time of interest, and fed into source analysis and synthetic planar gradient transformation. This transformation refers to a procedure whereby MEG data recorded with axial gradiometers is transformed as if it were measured by planar gradiometers (Bastiaansen and Knösche, 2000). The main advantage is that the spatial distribution of the resulting data is more readily interpretable.

For source analysis, we used a template anatomic scan provided by FieldTrip to create a volume conduction model based on a single shell model of the inner surface of the skull. The source model consisted of a regular grid spaced 0.5 cm apart that encompassed the entire brain. Leadfields were calculated and rank-reduced to two dimensions, to accommodate the fact that MEG is blind to tangential sources. The covariance of the data were calculated over the window of 1–8 s post-stimulus and regularized using shrinkage (Blankertz et al., 2011) with a regularization parameter of 0.05. The leadfields and data covariance were then used to calculate linearly constrained minimum variance spatial filters (LCMV, also known as beamformers; Van Veen et al., 1997). Applying these filters to sensor-level data yields activity estimates of a two-dimensional dipole at each grid point. We further reduced these estimates to a scalar value by means of the Pythagorean theorem. This leads to a positivity bias however, that we corrected for using a permutation procedure (Manahova et al., 2017). The number of permutations was 10,000. The final result was interpolated to be projected on a cortical surface and quantifies the degree to which a particular area contributed to the performance of the classifier/decoder.

## Results

### Behavioral results

The average accuracies in the MEG session for the four conditions ranged from 68% to 72%, confirming that subjects were able to do the task, as well as that the staircase procedure was successful. The average final jitter estimate from the staircase procedure for the 0°, 60°, 120°, and 180° conditions were as follows (95%-CI in parentheses): 3.1° (0.98-5.2), 11.0° (8.87-13.0), 13.4° (11.35–15.52), and 6.5° (4.42-8.60), respectively. With the exception of the 180° condition, the rising trend in these values suggests that subjects found the task more difficult when the amount of rotation was larger. The relatively low value for the 180° condition however indicates that this condition was relatively easy. One explanation may be the fact that subjects did not require the final product of the MR to perform well on the task. It is possible that they simultaneously memorized the starting orientation. After having finished the MR, regardless of how well they were able to do so, they could simply reactivate the initial image and use that in their judgment.

### Gaze position tracks VWM contents

**Figure 2.**
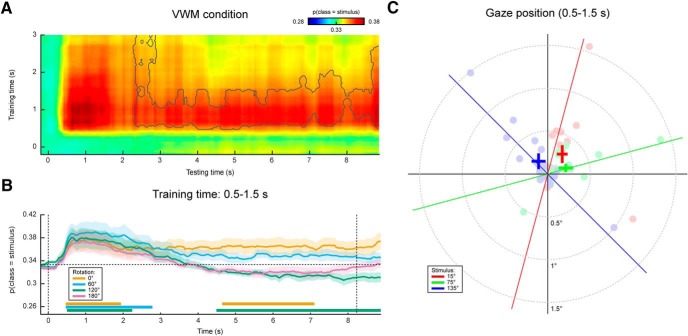
Gaze position classification results, cross-validation within VWM/imagery task. ***A***, Temporal generalization matrix of classification performance in the VWM condition. The color scale denotes the average posterior probability that the data belong to the same class as the presented stimulus. The gray outline demarcates a near-significant cluster (*p* = 0.069). Note that this matrix is asymmetric because only the VWM condition is shown, while the classifier was trained on the data from all VWM/MR conditions. For this reason, the data after ∼3 s are not expected to contain systematic patterns and therefore the training time axis has been truncated (Extended Data Fig. 2-1). ***B***, Classification performance averaged over the training time window of 0.5–1.5 s, separately for the VWM and the three MR conditions. Note that the 0° condition corresponds directly to the matrix in ***A***. The two vertical dashed lines indicate stimulus and probe onset. Shaded areas indicate the SEM. Significant clusters are indicated by the horizontal bars in the lower part of the figure. ***C***, Average gaze position during 0.5–1.5 s after stimulus onset, separately per stimulus orientation. Each transparent dot corresponds to an individual subject. The crosses are the grand averages, where the vertical and horizontal arms denote the SEM. The three colored lines depict the orientation of the three stimuli.

In the eye movement analysis, we investigated whether there is a relation between gaze position and the item held in VWM. We adopted the same analysis in our original main analysis (see Materials and Methods), but instead entered the horizontal and vertical gaze position, measured by the eye-tracker, as features in the decoding analysis. Specifically, we constructed a three-class probabilistic classifier that yields the posterior probabilities that any given data belong to either of three presented orientations. That is, the classifier was trained according to the labels of the presented stimulus. The classifier was trained on trials from all conditions (i.e., all amounts and directions of rotation) pooled together to obtain maximum sensitivity (for rationale, see Materials and Methods). To verify whether we could decode stimulus identity from the gaze position, we first applied the classifier to the same (pooled) data using cross-validation. We found above-chance decoding in a time period of ∼0.5–3.5 s post-stimulus that was marginally significant (Fig. 2, Extended Data Fig. 2-1). This indicates that subjects moved their eyes in a way consistent with the present stimulus, and kept it there for ∼2–3 s.

10.1523/ENEURO.0401-17.2018.f2-1 Extended Data Figure 2-1 Gaze position classification performance within the VWM/imagery task, pooled across VWM and MR conditions. Temporal generalization matrix of the average posterior probability that the data belong to the same class as the presented stimulus, pooled across all rotation conditions (see Materials and Methods). Note that the relatively short-term classification of approximately 3 s is expected, because the subjects rotate their mental image in clockwise direction on some of the trials and in counterclockwise on others. Hence, any reliable relation between the presented stimulus (the factor that the classifier was tested and trained on) and the mental image cancels out over the course of the delay interval. The gray outline corresponds to a near-significant cluster (*p* = 0.068). Download Figure 2-1, TIF file.

Then, when looking at the decoded signal within the VWM condition only, we found a sustained pattern (Fig. 2*A*), although again only marginally significant. This suggests that on perceiving and encoding the stimulus, subjects move their eyes in a way systematically related to the identity of the stimulus and keep that gaze position stable throughout the entire delay period.

**Figure 3.**
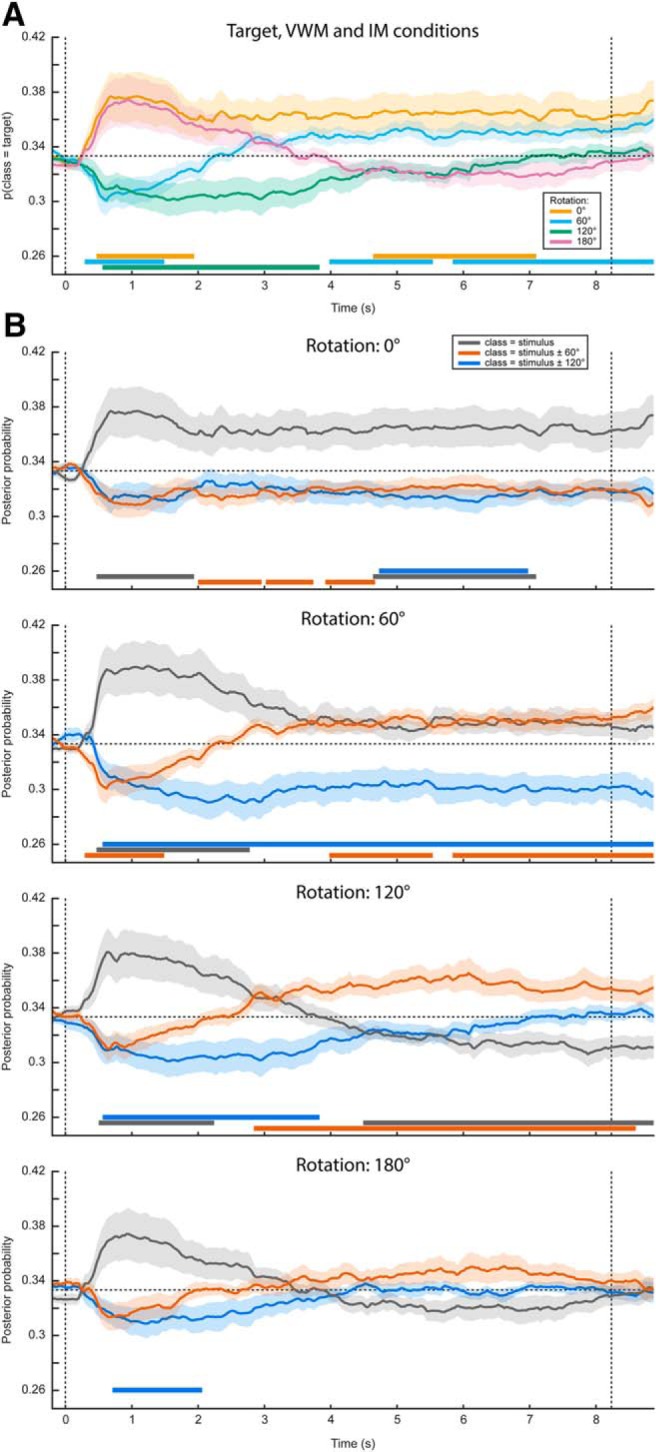
Complete gaze position classification results. Analogously to Figure 2, the classifier was trained on the time window of 0.5–1.5 s after stimulus onset, and according to the labels of the presented stimulus. ***A***, Average posterior probability that the data belong to the class of the target orientation, that is, the orientation that the subjects were supposed to have in mind at the end of the delay period. For both the 0° and the 180° conditions, the target orientation was the same as the presented stimulus. For the 60° and 120° conditions, however, the target and presented stimulus were different, hence the below-chance probabilities at the beginning of the delay period. ***B***, The average posterior probabilities that the data belong to either of three classes: the same orientation as the presented stimulus, the orientation of the presented stimulus ±60° or the orientation of the presented stimulus ±120°, plotted separately for the four VWM/MR conditions. The plus/minus sign is due to the MR being performed either clockwise or counterclockwise. This figure gives insight into whether the feature patterns corresponding to any intermediate orientations become active during MR, which is particularly relevant for the 120° and 180° conditions. For instance, in the 180° counterclockwise condition, the subject would start with a mental image with the same orientation as the presented stimulus, then pass through, respectively, -60° and -120°, ultimately to reach the target of -180° (i.e., 0°). If the gaze positions corresponding to all these orientations become active in sequence, one would first expect a peak in posterior probability that the data belong to the same class as the stimulus (gray line, bottom figure), then a peak in the probability of belonging to the presented stimulus -60° (orange line), then for -120° (blue line), and finally again for 0° (gray line). It is important to realize that below-chance probabilities in these analyses are meaningful. For instance, consider ***A***. Here, the probability that the data belong to the same class as the target is plotted. Hence, in the 60° and 120° conditions, the classifier correctly identifies that the data do not belong to the same class as the target in the beginning of the interval, because the starting orientation was different. As another example, consider the red line in the second panel in ***B***. This line plots the probability that the data belong to the starting orientation ±60°. On presentation of the starting orientation, the classifier therefore yields a significant below-chance probability. However, as the subject performs the ±60° rotation over the course of the trial, the classifier increasingly picks up this rotated image, hence giving above-chance probabilities. Note that ***A***, ***B***, as well as Figure 2, all depict the same data but visualized in different manners. Shaded areas denote the SEM and significant clusters are depicted by the thick horizontal lines at the bottom of the panels.

Contrary to previously used paradigms, where two stimuli were displayed at the beginning of a trial and a retro-cue signaled the item that was to be remembered (Harrison and Tong, 2009; Albers et al., 2013; Christophel et al., 2015, 2017), in the present experiment we only showed one stimulus. It is therefore possible that the sustained decoding performance does not necessarily reflect VWM contents, but simply that the subjects moved their gaze according to the presented stimulus rather than to their mental contents. However, if this were true, then we should find a similar effect in the three MR conditions. If, on the other hand, the classifier picked up the item kept in mind, then the probability that a trial is assigned to the same class as the presented stimulus should drop over time, as the subject rotates the mentally imagined grating away from the starting orientation. Our results were consistent with the latter scenario (Fig. 2*B*). Whereas the probability that the data belong to the same class as the presented stimulus stays steadily above chance in the VWM condition, it drops to lower levels in the three MR conditions. Moreover, we found evidence that the gaze moves toward a position consistent with the orientation of the presented grating plus or minus 60° (depending on the cued direction of rotation) in the MR conditions, but not any further (Fig. 3*A*,*B*).

Figure 2*C* displays the grand average, as well as individual average gaze positions during 0.5–1.5 s after stimulus onset, separately for each of the three stimulus conditions, collapsed across VWM and MR conditions. Although there is large variability among subjects in the magnitude of the eye movements, a general trend can be discerned where subjects position their gaze along the orientation axis of the grating. The mean disparity in visual angle with respect to pre-trial fixation was only 0.23°, which is in the same order of magnitude as reported previously (Foster et al., 2016), although for some subjects, it was larger, up to 1.5°.

In short, there was a systematic relationship between gaze position and stimulus orientation, after which the gaze position tracked the orientation kept in mind during the delay period, but only for a maximum of approximately ±60° relative to starting orientation. These findings raise the concern that any potential decoding of VWM items from MEG signals, as was the aim of our original analysis, could be the result of stimulus-related eye confounds (for possible underlying mechanisms, see Discussion).

### Sustained decoding of VWM items from MEG signals

**Figure 4.**
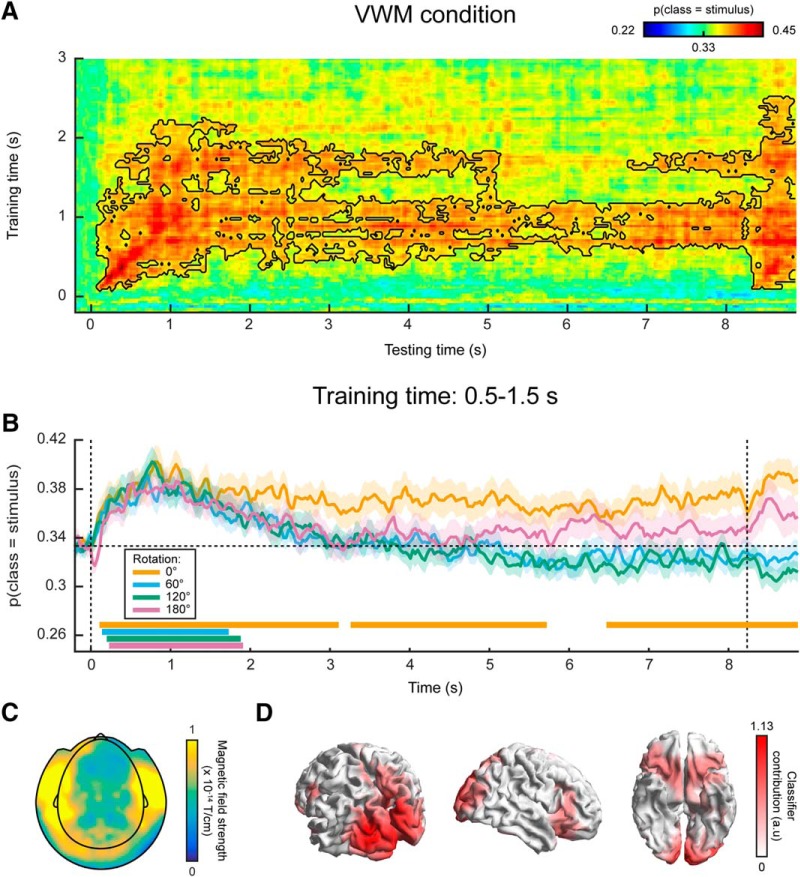
MEG classification results. ***A***, Same as in Figure 2*A*, except the classification was performed on MEG data, rather than on gaze position. The black outline demarcates a significant cluster (*p* = 0.006). ***B***, Same as in Figure 2*B*, except the analysis was performed on MEG data. Synthetic planar gradiometer topography (***C***) and source topography (***D***) of areas that contributed to the classifier. See also Extended Data Figure 4-1.

The original aim of this study was to assess the representational contents of the neural signals while the subjects were engaged in VWM/imagery. We present these results here, to demonstrate how they could easily have been mistaken for genuine results, had we been oblivious to the systematic eye movements. We constructed a three-class probabilistic classifier in which the MEG sensors were entered as features. As before, the classifier was trained on trials from all conditions (i.e., all amounts and directions of rotation) pooled together for maximum sensitivity (for rationale, see Materials and Methods). To test whether we could decode stimulus identity from the MEG signal, we applied the classifier to the same (pooled) data using cross-validation and found successful decoding during a period of up to ∼2.5 s after stimulus onset (Fig. 4, Extended Data Fig. 4-1). The stimulus itself was presented for only 250 ms. Therefore, the later part of this period could have be interpreted as an endogenous representation, for instance stemming from active mental instantiation by the subject, although in reality it is more likely to be the result of eye movements.

10.1523/ENEURO.0401-17.2018.f4-1 Extended Data Figure 4-1 MEG classification performance within the VWM/imagery task, pooled across VWM and MR conditions. Similar to Extended Data Figure 2-1, except the classifier is trained and tested on MEG data rather than on gaze position. Black outline indicates a significant cluster (*p* = 0.007). Download Figure 4-1, TIF file.

Next, we looked at the decoding performance in the VWM condition alone, using the classifier trained on all conditions as described above. We found that the identity of the memory item could be decoded during the entire delay interval, using classifiers obtained from a training window of ∼0.5–1.5 s (Fig. 4*A*). The performance stayed above chance-level (33.3%) at a stable level of ∼37% throughout the entire interval (Fig. 4*B*).

Again, we found this sustained pattern to be specific to the VWM condition, because the probability that the data belong to the same class as the presented stimulus drops over time in the three MR conditions (Fig. 4*B*). As explained in the previous paragraph, this indicates that the sustained above-chance classification in the VWM condition cannot be explained as a long-lasting stimulus-driven effect (e.g., stimulus aftereffect), but must also reflect the memorized item to at least some degree. In the 180° condition, the posterior probability that the data belong to the same classes as the presented stimulus later reemerges as a rising, although nonsignificant trend. This can be explained by the fact that the final orientation that the subjects should have in mind in the 180° condition is identical to the orientation of the presented stimulus at the start of a trial.

To facilitate interpretation of these results, we inspected the classifier’s corresponding sensor topography (Fig. 4*C*) and source localization (Fig. 4*D*), averaged over the training time period of 0.5–1.5 s. These indicate that both occipital (Harrison and Tong, 2009; Albers et al., 2013) and prefrontal sources (Sreenivasan et al., 2014; Spaak et al., 2017) contributed to the classifier’s performance. Indeed, the prefrontal sources could in reality point to ocular sources. Moreover, although the contribution from occipital regions may seem to provide evidence that the decoder genuinely picks up visual representations, these sources could in fact also be driven by the eye movements (see Discussion).

**Figure 5.**
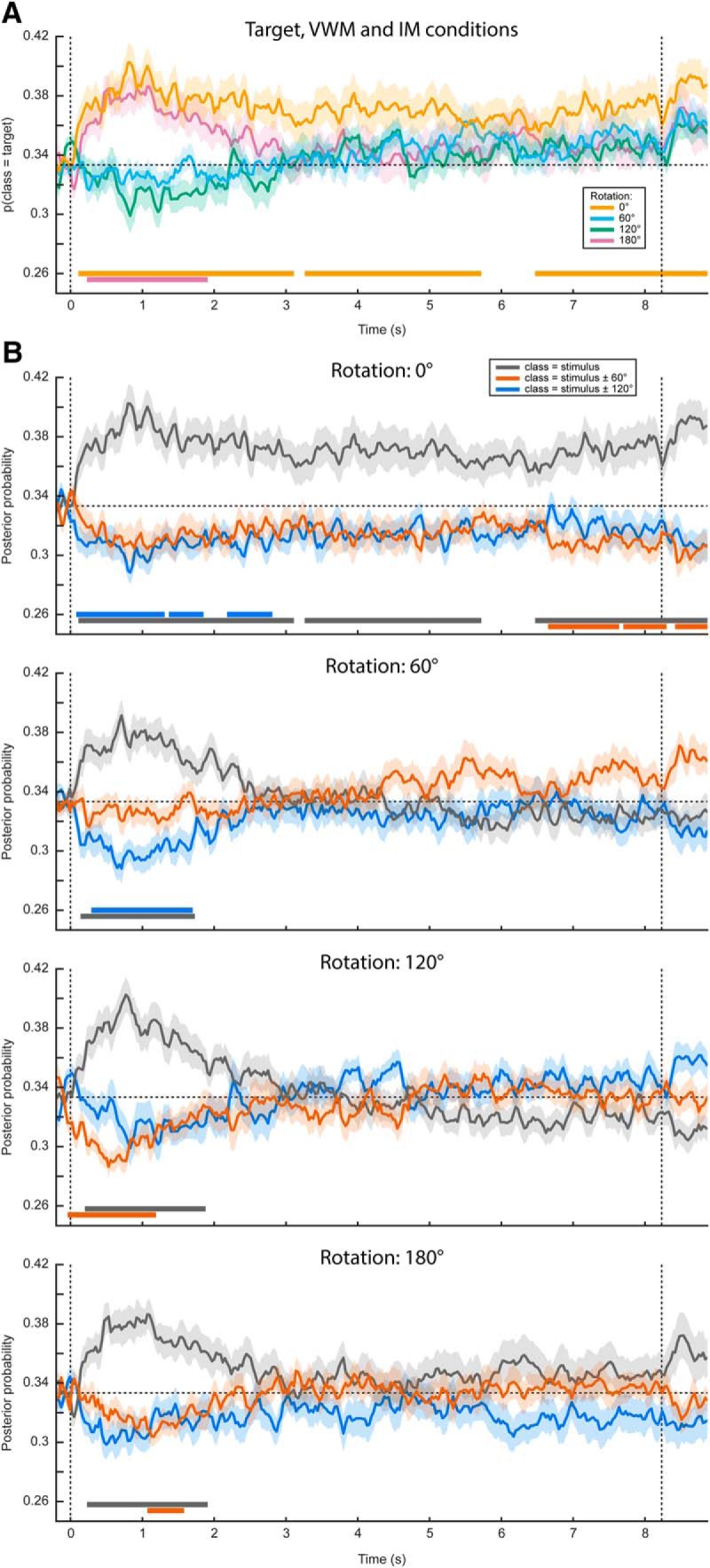
Complete MEG classification results, visualized in a variety of ways. This figure is analogous to Figure 3, except the classifier is trained and tested on MEG data rather than on gaze position.

Finally, we investigated whether we could decode the intermediate (for the 120° and 180° rotations) and the final orientations in the MR conditions. We found some indication that the final orientation, but not the intermediate ones (Fig. 5*B*), indeed emerges halfway through the delay period, but this effect was not statistically significant (Fig. 5*A*).

### Decoding visual representations from sensory areas

**Figure 6.**
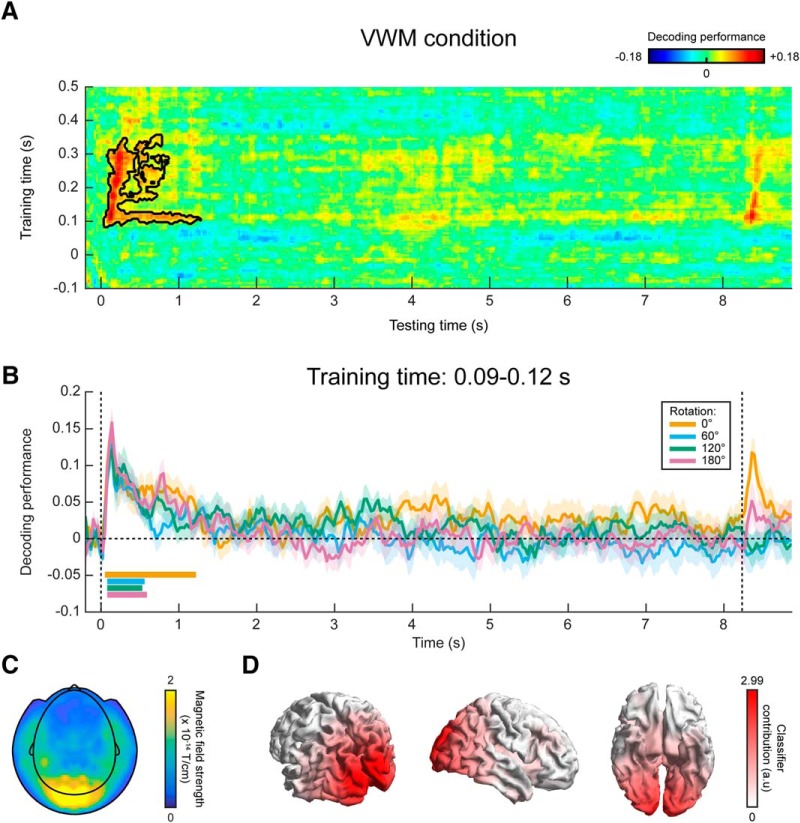
MEG decoding results, generalized from localizer to VWM/imagery task. ***A***, Temporal generalization matrix of orientation decoding performance, for which the decoder was trained on all time points in the functional localizer and tested across all time points in the VWM/imagery task. The color scale reflects the correspondence between true and decoded orientation. The black outline shows a significant cluster (*p* = 0.04). Note that the *x*- and *y*-axis in the figure are differently scaled for optimal visualization. ***B***, Decoding performance over time in the VWM/imagery task, averaged over decoders trained in the window of 0.09–0.12 s in the localizer, separately for the VWM and three MR conditions. Shaded areas denote the SEM, and significant clusters are indicated by the horizontal bars. Synthetic planar gradiometer topography (***C***) and source topography (***D***) of areas that contribute to the decoder. See also Extended Data Figures 6-1, 6-2.

In a third analysis, we trained a continuous orientation decoder (see Materials and Methods) on the functional localizer data (Fig. 1*B*,*C*) and applied these decoders to the data from the VWM/imagery task (King and Dehaene, 2014). The main advantage of this method is that it ensures that the decoder is primarily sensitive to sensory signals, and not to higher-level top-down processes involved in mental manipulation of an image. It thus allows us to track sensory-specific activation throughout the delay period (Mostert et al., 2015). Cross-validation within the functional localizer confirmed that we were indeed able to reliably decode orientation-specific information from activity evoked by passively perceived gratings (Fig. 6, Extended Data Fig. 6-1). Moreover, we were not able to decode grating orientation on the basis of gaze position, verifying that the data from the functional localizer were not contaminated by stimulus-specific eye movements (Extended Data Fig. 6-2).

10.1523/ENEURO.0401-17.2018.f6-1 Extended Data Figure 6-1 MEG decoding performance within the functional localizer using cross-validation. The time axis represents matched training and testing time points. Shaded areas denote the SEM, and the horizontal line demarcates a significant cluster (*p* = ±0). Download Figure 6-1, TIF file.

10.1523/ENEURO.0401-17.2018.f6-2 Extended Data Figure 6-2 Gaze position classification performance within the functional localizer, using cross-validation. ***A***, The time axis represents matched training and testing time points. No significant above-chance classification was found. Note that although there appears to be a rise in performance after approximately 500 ms, this only reached a value of 0.1675 at its peak (*t* = 0.51 s), which is very little above chance (0.1667 for six classes). Shaded areas denote SEM. ***B***, Average gaze position at 0.51 s after stimulus onset, separately per stimulus orientation. Each transparent dot corresponds to an individual participant. The crosses are the grand averages, where the vertical and horizontal arms denote the SEM. The six colored lines depict the orientation of the six stimuli. Download Figure 6-2, TIF file.

In the VWM condition, the decoders trained on the functional localizer data could reliably decode the orientation of the presented stimulus (Fig. 6*A*). Moreover, for a training time of ∼90–120 ms, we could decode the stimulus for a prolonged time, lasting over 1 s after stimulus onset. Interestingly, this training time point coincides with the time at which peak performance is obtained within the localizer itself (Extended Data Fig. 6-1). Comparing the decoding trace within this training time window with the three MR conditions, it can be seen that grating orientation can be decoded in all four conditions for a sustained period of ∼500 ms (Fig. 6*B*). This is in sharp contrast to the extended decoding of the presented stimulus throughout the entire delay period, found within the VWM/imagery task using cross-validation (Fig. 4).

We inspected the spatial pattern (Fig. 6*C*) and corresponding source topography (Fig. 6*D*) for the decoders, averaged across training time 90–120 ms. These highlight primarily occipital regions as contributing to the decoder’s performance, consistent with our premise that the functional localizer primarily induced bottom-up sensory signals, especially during this early time interval (Mostert et al., 2015).

In summary, our findings suggest that the stable, persistent representation found in our within-task MEG decoding result may well be attributed to stimulus-specific eye movements, although the magnitude of the eye movements were only small. In contrast, no clear evidence was found for such a long-lasting representation when training the decoder on the functional localizer. Given that the localizer was not contaminated by stimulus-specific eye movements, these results thus provide a more reliable picture of the sensory representations during the delay interval.

## Discussion

Neural decoding is a powerful and promising technique for neuroimaging studies (Haxby et al., 2014; Grootswagers et al., 2017) that has led to substantial advancement in the field of VWM over recent years (Harrison and Tong, 2009; Serences et al., 2009; Albers et al., 2013; Wolff et al., 2015, 2017). This study also employed neural decoding techniques, with the original aim to investigate the temporal dynamics of sensory representations during VWM. However, we found that our data were contaminated by small but systematic eye movements, whereby gaze position was related to the stimulus held in mind. This jeopardized our ability to interpret the neural decoding results, results that otherwise would have seemed sensible, because very similar results could be obtained by considering gaze position only.

There are at least three possible mechanisms via which stimulus-specific eye movements may confound our results. First, eye movements are known to cause stereotypical artifacts in MEG recordings. Due to the positively charged cornea and negatively charged retina, the eyeball acts as an electromagnetic dipole whose rotation is picked up by the MEG sensors. The spatial pattern that the dipole evokes is directly related to its angle, or in other words, to the position of the subject’s gaze (Plöchl et al., 2012). Thus, if the subject moves his/her eyes in response to the grating in a manner related to the orientation of that grating, then this will induce a specific pattern in the MEG signals, which in turn is directly related to the grating orientation. A decoding analysis applied to these signals is then likely to pick up the patterns evoked by the eyeball dipoles, confounding potential orientation-related information stemming from genuine neural sources. In fact, our source analysis hints at this scenario (Fig. 4*D*), as the contributions from presumed prefrontal sources closely resemble an ocular source.

Second, if the eyes move, then the projection falling on the retina will also change, even when external visual stimulation remains identical. Thus, if gaze position is systematically modulated by the image that is perceived or kept in mind, then so is the visual information transmitted to the visual cortex. For example, if a vertical grating is presented and kept in VWM, then the subject may subtly move her or his gaze upward. Correspondingly, the fixation dot is now slightly below fixation, thus leading to visual cortex activity that is directly related to the retinotopic position of the fixation dot. Our decoding analysis may thus actually decode the position of the fixation dot, rather than grating orientation, potentially leading to an incorrect conclusion. Source analysis would in this scenario also point to occipital sources, similarly to what we found (Fig. 4*D*). Note that this mechanism is not specifically dependent on the presence of a fixation dot. A systematic difference in eye position will also lead to changes in the retinotopic position of, for instance, the presentation display or the optically visible part of the MEG helmet.

Third, if gaze position covaries with the mental image, then decoding of the mental image will also reveal areas that encode eye gaze position, such as oculomotor regions in parietal and prefrontal cortex.

Our findings raise the question of why there were task-induced eye movements that were directly related to the grating kept in VWM. In fact, there is a considerable mass of literature that describes the role of eye movements in mental imagery. It has been found that subjects tend to make similar eye movements during imagery as during perception of the same stimulus (Brandt and Stark, 1997; Laeng and Teodorescu, 2002; Laeng et al., 2014). Already proposed by Donald Hebb (Hebb, 1968), it is now thought that eye movements serve to guide the mental reconstruction of an imagined stimulus, possibly by dwelling on salient parts of the image (Spivey and Geng, 2001; Laeng et al., 2014). Moreover, the specificity of the eye movements is also related to neural reactivation (Bone et al., 2017) and recall accuracy (Laeng and Teodorescu, 2002; Laeng et al., 2014; Bone et al., 2017). Our findings are in accordance with these studies. Subjects’ gaze was positioned along the orientation axis of the grating - that is, the visual location within the stimulus that provided the highest information regarding its orientation and is thus exactly what one would expect given that the task was to make a fine-grained orientation comparison with a probe grating. Importantly, however, subjects were explicitly instructed to maintain fixation throughout the entire trial. We nevertheless observed that not all subjects adhered to this requirement, albeit involuntarily.

Despite these problems associated with the systematic eye movements in our experiment, it is still possible that our decoding results do in reality stem from genuine orientation information encoded in true neural sources. In fact, we used independent component analysis in our pre-processing pipeline to (presumably) remove eye-movement artifacts. However, it would be very difficult, if not impossible, to convincingly establish that no artifacts remain and, considering the similarities between the decoding results from the MEG data (Fig. 4*A*,*B*) and the gaze position (Fig. 2*A*,*B*), we feel any attempts at this would be unwarranted.

Given the potential pervasiveness of systematic eye movements in VWM/imagery tasks, and the demonstrated susceptibility of our analysis methods to these confounds, one wonders whether other studies may have been similarly affected. Clearly, the first mechanism described above involving the eyeball dipole would only affect electrophysiological measurements like electroencephalography and MEG, and has indeed been a concern in practice (Foster et al., 2016). The second mechanism however, whereby stimulus identity is confounded with the retinal position of visual input, would also affect other neuroimaging techniques such as fMRI. This confound could be particularly difficult to recognize, because it would also affect activity in visual areas. Moreover, because eye movements during imagery have been found to be positively related to performance (Laeng and Teodorescu, 2002), this could potentially explain correlations between VWM decoding and behavioral performance. The third mechanism, whereby one directly decodes gaze position from motor areas, could be a problem especially for fMRI which, thanks to its high spatial resolution, might be well able to decode such subtle neural signals. This concern may be especially relevant for studies that investigate the role of areas involved in eye movements or planning thereof, such as frontal eye fields or superior precentral sulcus, in the maintenance of working memory items (Jerde et al., 2012; Ester et al., 2015; Christophel et al., 2017).

This leaves the question of how to deal with eye movements in VWM/imagery tasks. Naturally, it is important to record eye movements during the experiment, for instance using an eye-tracker or EOG. One can then test for any systematic relationship and, if found, investigate whether it could confound the main results. In our case, for example, decoding of gaze position leads to strikingly similar results as those obtained from the MEG data. Foster et al. (2016) on the other hand found that decoding performance of working memory items decreased throughout the trial, whereas the deviation in gaze position increased, suggesting that eye confounds cannot explain the main findings. Another approach might be to design the experimental task in such a way that eye movements are less likely. For example, by presenting gratings laterally (Pratte and Tong, 2014; Ester et al., 2015; Wolff et al., 2017), and assuming that VWM items are stored in a retinotopically specific manner (Pratte and Tong, 2014), the involuntary tendency to move one's eyes subtly along the remembered grating's orientation axis may become less strong, because those gratings are located distantly from the gaze’s initial location (i.e., central fixation). Finally, a powerful approach could be to adopt a separate functional localizer, which allows specific decoding of functionally defined representations such as bottom-up, sensory-specific signals (Harrison and Tong, 2009; Serences et al., 2009; Albers et al., 2013; Mostert et al., 2015). If the localizer is well designed and not systematically contaminated by eye movements, then eye movements in the main task cannot have a systematic effect on the decoded signal, thus effectively filtering them out.

We designed a localizer that is specifically sensitive to the neural representations encoded in bottom-up signals evoked by passively perceived gratings. This allowed us to address the question of whether the imagined stimulus was encoded with a similar neural code as the perceived gratings (Harrison and Tong, 2009; Albers et al., 2013). Using this localizer, we indeed obtained MEG decoding results that were very dissimilar from those obtained using cross-validation within the combined VWM/imagery task. We no longer found persistent activation of an orientation-specific representation throughout the entire delay period. Nevertheless, the sensory pattern did remain above baseline for a period of ∼1 s, which is relatively long considering that the stimulus was presented for only 250 ms. One explanation is that the stimulus was relevant for the task. Previous work has shown that task relevance may keep the sensory representation online for a prolonged period even after the stimulus is no longer on the screen (Mostert et al., 2015).

It should be pointed out that using a functional localizer also has its intrinsic limitations. The most important being that, while such an approach is primarily sensitive to a functionally defined signal, it may at the same time be blind to other relevant signals that were not a priori included in the functional definition. The VWM literature itself provides an instructive example: while the functional localizer approach has clearly demonstrated sensory representations of the memorandum in associated sensory cortex (Harrison and Tong, 2009; Albers et al., 2013), it would have missed relevant encoding in other regions in the brain such as parietal and prefrontal cortex (Christophel et al., 2015, 2017). Furthermore, the fact that the decoders were trained on a functionally defined signal does not mean that they are necessarily insensitive to other signals, such as eye movement-related signals. However, it is important to realize that the exact effect of these other signals on the decoder’s output is not explicitly defined. These potential effects would therefore be idiosyncratic to an individual’s data and are expected to cancel out at the group level.

In summary, we demonstrate a case where decoding analyses in a VWM/imagery task are heavily confounded by systematic eye movements. Given the high potential benefit of decoding analyses and its widespread use in the study of working memory and mental imagery, we argue that this problem may be more pervasive than is commonly appreciated. Future studies could target this question specifically and investigate how strong the confounds are exactly. One approach could be to systematically vary salient input and assess how this impacts decoding performance (cf. the second mechanism described above). Furthermore, it is important to realize that this does not necessarily invalidate all previous studies. While some previous results may have been afflicted, our current understanding of the neural underpinnings of VWM is still firmly grounded in converging evidence from a wide variety of techniques, paradigms and modalities. Nevertheless, we conclude that eye movement confounds should be taken seriously in both the design as well as the analysis phase of future studies.

## Synthesis

Reviewing Editor: Satu Palva, University of Helsinki Decisions are customarily a result of the Reviewing Editor and the peer reviewers coming together and discussing their recommendations until a consensus is reached. When revisions are invited, a fact-based synthesis statement explaining their decision and outlining what is needed to prepare a revision will be listed below. The following reviewer(s) agreed to reveal their identity: Lauri Parkkonen.

Synthesis:

Overall, both reviewers found your study very interesting and timely and to contribute novel knowledge regarding the role of eye-movements in visual working memory. Also the experiment, analysis and statistics were found to be sound. However, as a major issue that should be corrected before publication it was found that the structure of the manuscript was centered along the working memory framework while the main findings was the role of eye-movements in working memory. Therefore, it was felt that before publication, the manuscript should be restructured and interpretation of the results should be modified along these lines. I have attached below some of the reviewer comments.

***

Reviewer # 1

Major

- I wonder whether there might not be a better large-scale structure for the manuscript. Currently, the authors wrote the manuscript “as if” this was a study on VWM (both in introduction, and in results, and in discussion), to then present “controls” which in their view, however, invalidate their results. Is this manuscript not about eye-movement related confounds in neural decoding of VWM (as the title says), rather than about the nature of VWM? Then should not those be the main topic of the manuscript?

- Although I believe the authors treat the issue of a potential confound which might invalidate a large number of previous studies without alarmism and with the proper care, I still have doubt that this manuscript will be received positively by the VWM community - while there is no direct attack on the studies enumerated e.g. in the introduction, transitively they are put into question. This is merely an invitation to once again seriously consider the exposition of the results with an eye of how an uncharitable reader might see this, and whether this is in the best interest of the community, or the first author. The aim is to avoid provoking negative reactions within the scientific society that might render it unable to appreciate the importance of the reported findings.

- I suggest including Fig 3-2 into the main manuscript. The amount of data the authors present to argue for eye movements as a driver of VWM content decoding is not very rich, and adding this information can remedy that. I further suggest including Fig. 4 in the main manuscript for similar reasons.

- Fig. 1 A reports temporal generalization results that are asymmetric for training and testing time. The authors need to comment on why this is the case (e.g. how SNR of the training set affects the results, or whether mental representations at one time point are a sub/ or supra-set of mental representations at another time point).

- There seems to be a contradiction in the author's treatment of the behavioral data (rotation-related jitter) and the results of the decoding (Fig. 1B). On the one hand from the low jitter (behavioral experiment) for the 180degrees condition the authors speculate that participants did not rotate the grating mentally. About the decoding in Fig 2B, that state that it makes sense it goes down to 0 (as the other rotated orientations) to then rise above 0 (when presumably participants have finished rotating the stimulus that is then identical to the non-rotated one). Please clear this up.

- In the eye movement decoding results there are a lot of decoding accuracies significantly below chance (Fig. 3-2). Might there be the case that the classifier is reliably guessing that the examples come from opposite classes as they actually do? Could the authors please explain these results and state in the paper that these below chance significant time points are not included in the main inference about significance level of stimulus-specific eye movement decoding?

- On the one hand, the authors conclude from their results that “the maintenance of the memory item is described by the same neural code as employed in presumed mental re-instantiation of the stimulus shortly after its presentation, but not by the neural code evoked by the initial presentation of the stimulus”. On the other hand, to avoid eye confounds, authors apply a decoder trained on data from perception task to VWM task because this decoder “is sensitive to sensory signals only, and not to higher-level top-down processes involved in mentally manipulating an image”. If it holds true that this decoder excludes the majority of top-down influences, it could have excluded any possibility for VWM reactivation via attention-driven influences or any other brain-related (but eye movement-unrelated) top-down signals which could be crucial for WM maintenance. Is the perception localizer anyhow beneficial then for studying WM processes if it potentially cuts them off?

Minor

- l. 47: Is “latent variable” really a good term here?

- L 139-143 the two sentences say the same

- L 152-153 “only” is too strong, I suggest “predominantly”

- L 324 -329 the authors observe an increase in jitter estimate with increasing amount of rotation required. They do not provide a statistical test, though. The interpretation they offer makes sense, but we are only talking about 4 numbers, of which the authors find a trend only in 3 cases.

- Fig. 2D - Could the authors please specify the position of the localized sources more specifically, and relate them to previous literature on VWM? This manuscript highlights a confound that may be present in many neuroimaging studies that apply visual stimuli and multivariate pattern analysis, or decoding. Making the community aware of the possibility of such a confound is important.

Reviewer # 2

Major

My main concern regarding the manuscript comes from its likely history; it seems that the authors were first pursuing a visual working memory study but at a late stage realized that their results are possibly, or even likely, due to eye movements. Such a course of action shows scientific integrity and is therefore only to be appreciated but the structure of manuscript, and much of its text, still reflect the original goal of the study, which makes it unnecessarily difficult to grasp the current main message.

The current Results is a mixture of methods, actual results and discussion and appears to be in the style of some other journals which do not have a methods section before results. I would recommend making Results more compact.

In addition, to optimally address the possibility of eye-movement confounds, one could have designed control conditions such as having no fixation cross during the retention period (cf. the second point in Discussion, line 501 onward) and study how much the absence of salient visual input affects the results.

Minor

L10: “... working-memory tasks have been ...”

L84: The paragraph starts as if it was the first (cf. L65)

L107: Does your IRB really approve giving course credits for being a subject?

L140-142: The same idea presented twice

L216: Explain in which sense continuous

L223: Indicate the name of the classifier, if any

L271: Shouldn't you compare against an empirical chance level?

L286: “planar transformation” is jargon and not generally understood; please be more explicit about what was done

L316: Probably “help” < “kept”

L369: What is meant by “below chance level”?

L432: The concept “sensory-specific” is unclear. Consider something like “signals from sensory projection areas”

L457: A non-significant result is not a result; thus, I would recommend removing this sentence.

L650&656: The family name “de Lange” not correctly indicated

L714&768: Since the employed MEG system does not have real planar gradiometers, it would be better to use a phrase like “Planar gradient strength”

Fig. 2 & 3: Chance level could be indicated in the color bar and the same color scaling could be used across figures.
