# Supplementary figures and images for: Eye Movement-Related Confounds in Neural Decoding of Visual Working Memory Representations
Source: eNeuro. 2018 Oct 10;5(4):ENEURO.0401-17.2018. doi: 10.1523/ENEURO.0401-17.2018 (PMC6179574; doi:10.1523/ENEURO.0401-17.2018)

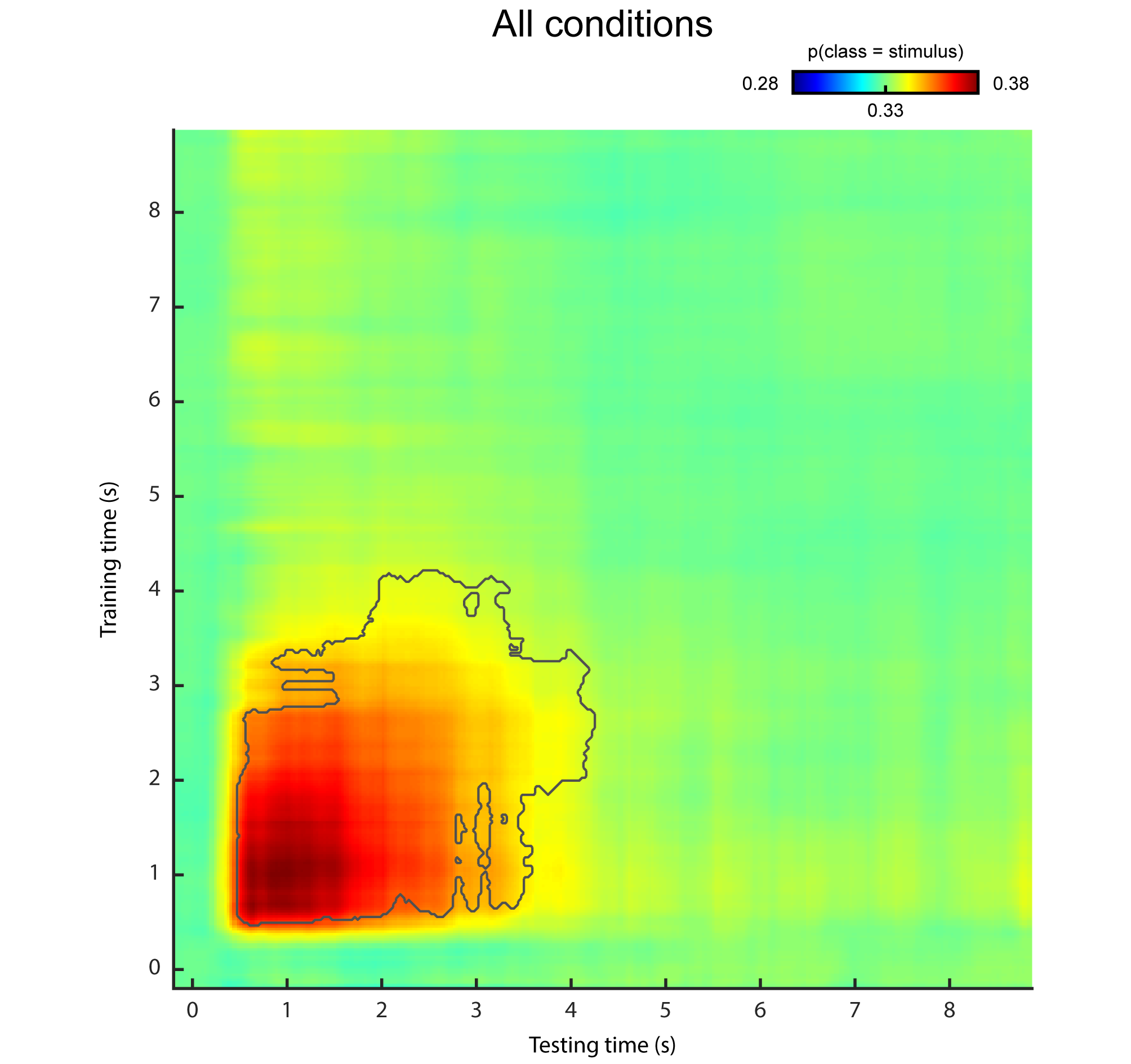

Supplement: Extended Data Figure 2-1 — Gaze position classification performance within the VWM/imagery task, pooled across VWM and MR conditions. Temporal generalization matrix of the average posterior probability that the data belong to the same class as the presented stimulus, pooled across all rotation conditions (see Materials and Methods). Note that the relatively short-term classification of approximately 3 s is expected, because the subjects rotate their mental image in clockwise direction on some of the trials and in counterclockwise on others. Hence, any reliable relation between the presented stimulus (the factor that the classifier was tested and trained on) and the mental image cancels out over the course of the delay interval. The gray outline corresponds to a near-significant cluster (p = 0.068). Download Figure 2-1, TIF file. [file sup_enu-eN-NWR-0401-17-s01.tif]

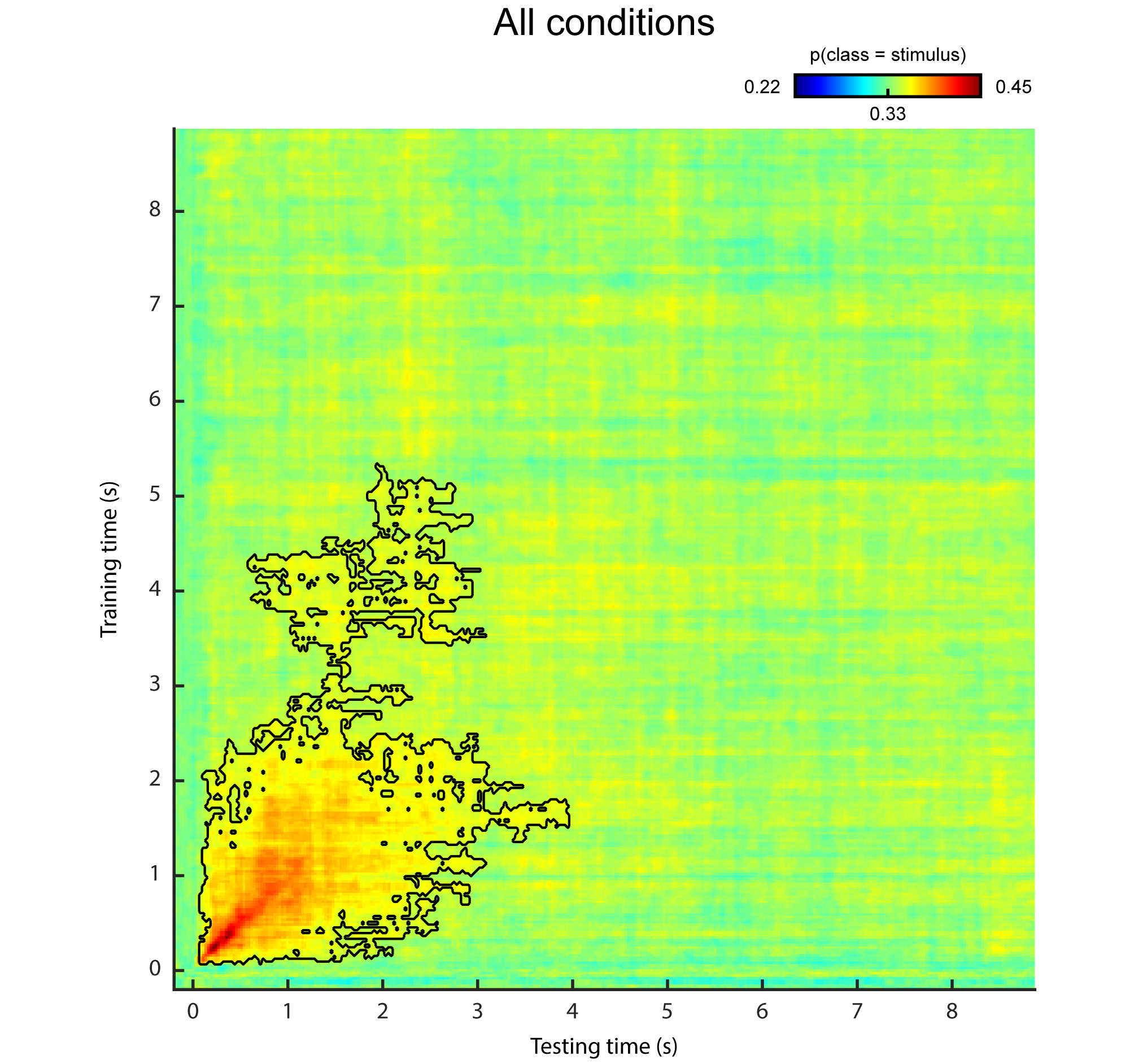

Supplement: Extended Data Figure 4-1 — MEG classification performance within the VWM/imagery task, pooled across VWM and MR conditions. Similar to Extended Data Figure 2-1, except the classifier is trained and tested on MEG data rather than on gaze position. Black outline indicates a significant cluster (p = 0.007). Download Figure 4-1, TIF file. [file sup_enu-eN-NWR-0401-17-s02.tif]

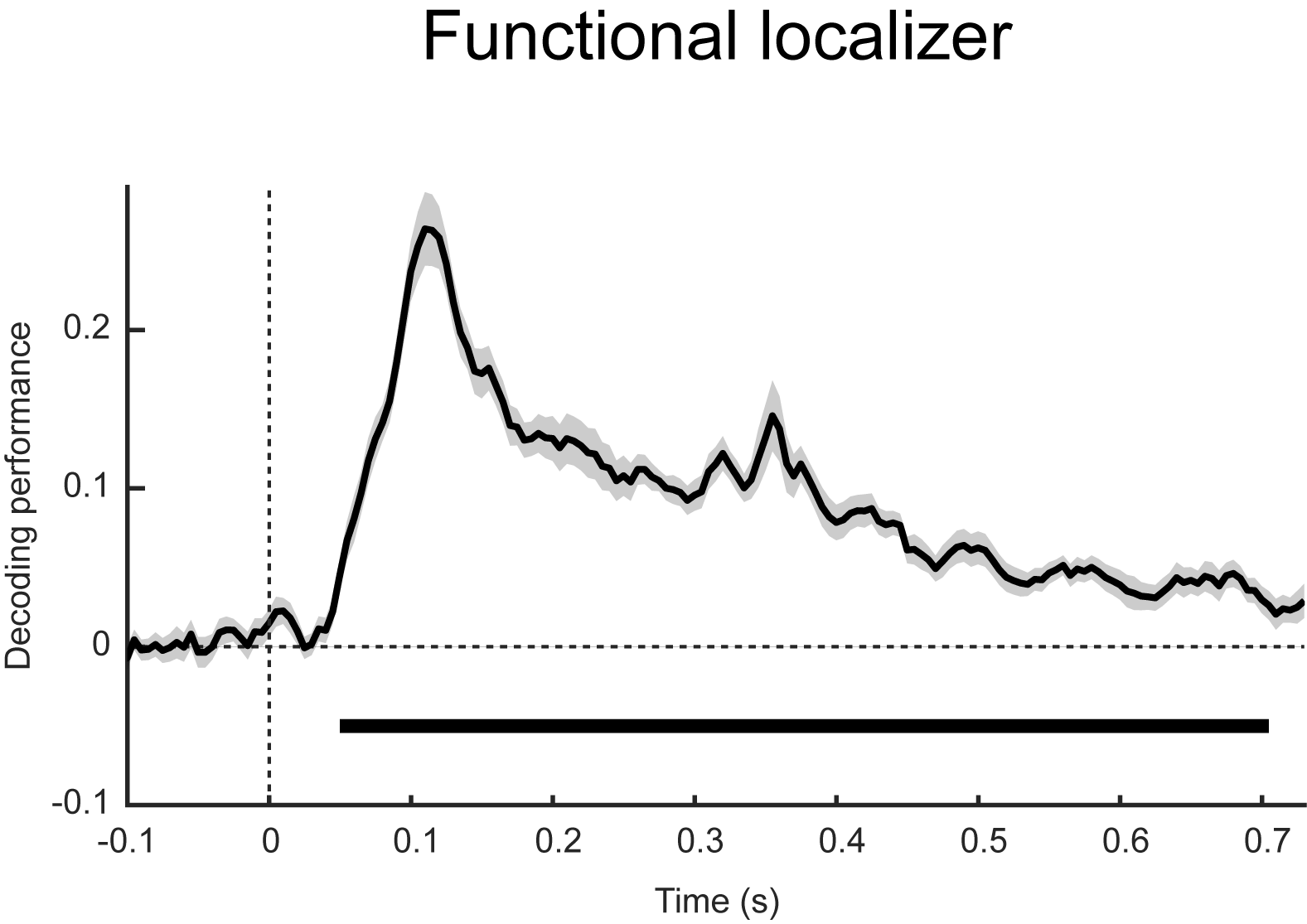

Supplement: Extended Data Figure 6-1 — MEG decoding performance within the functional localizer using cross-validation. The time axis represents matched training and testing time points. Shaded areas denote the SEM, and the horizontal line demarcates a significant cluster (p = ±0). Download Figure 6-1, TIF file. [file sup_enu-eN-NWR-0401-17-s03.tif]

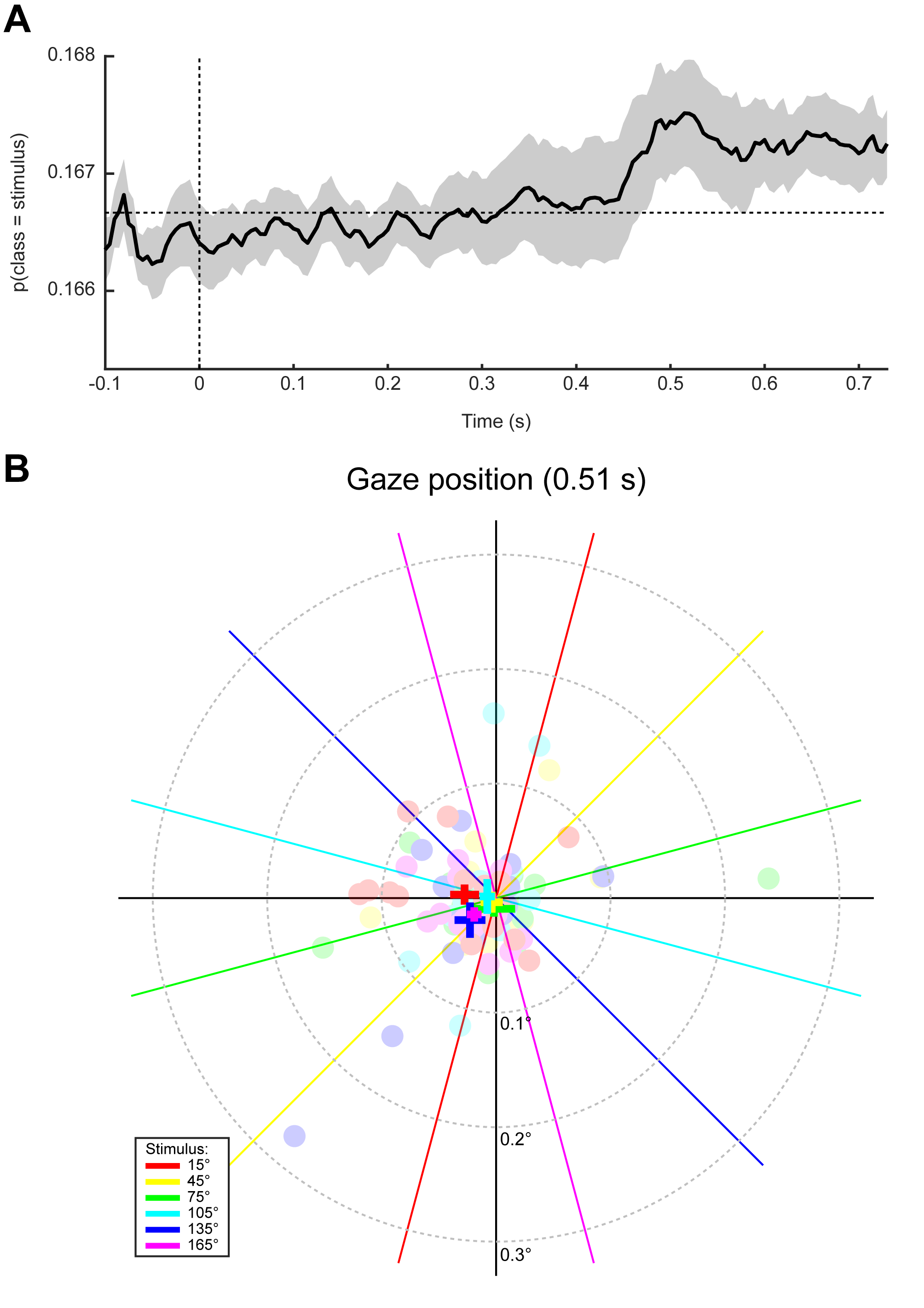

Supplement: Extended Data Figure 6-2 — Gaze position classification performance within the functional localizer, using cross-validation. A, The time axis represents matched training and testing time points. No significant above-chance classification was found. Note that although there appears to be a rise in performance after approximately 500 ms, this only reached a value of 0.1675 at its peak (t = 0.51 s), which is very little above chance (0.1667 for six classes). Shaded areas denote SEM. B, Average gaze position at 0.51 s after stimulus onset, separately per stimulus orientation. Each transparent dot corresponds to an individual participant. The crosses are the grand averages, where the vertical and horizontal arms denote the SEM. The six colored lines depict the orientation of the six stimuli. Download Figure 6-2, TIF file. [file sup_enu-eN-NWR-0401-17-s04.tif]
